# From the culture broth to the erythritol crystals: an opportunity for circular economy

**DOI:** 10.1007/s00253-021-11355-2

**Published:** 2021-05-27

**Authors:** Laura Daza-Serna, Sebastián Serna-Loaiza, Audrey Masi, Robert Ludwig Mach, Astrid Rosa Mach-Aigner, Anton Friedl

**Affiliations:** 1 Christian Doppler Laboratory for Optimized Expression of Carbohydrate-active Enzymes, Research Division Biochemical Technology, Institute of Chemical, Environmental and Bioscience Engineering, TU Wien, 1060 Vienna, Austria; 2 Research Unit of Bioresource and Plant Science, Institute of Chemical, Environmental and Bioscience Engineering, TU Wien, 1060 Vienna, Austria; 3 Research Unit of Biochemical Technology, Institute of Chemical, Environmental and Bioscience Engineering, TU Wien, 1060 Vienna, Austria

**Keywords:** Erythritol, Biotechnology, Downstream, Sustainable substrates, Circular economy

## Abstract

**Abstract:**

The reduction of sugar intake by adults has been stated by the World Health Organization as an important strategy to reduce the risk of non-communicable diseases. Erythritol is a four-carbon sugar alcohol that is considered as a highly suitable substitution for sucrose. This review article covers approaches for the separate stages of the biotechnological production of erythritol from cultivation to the downstream section. The first part focuses on the cultivation stage and compares the yields of erythritol and arising by-products achieved with different types of substrates (commercial versus alternative ones). The reported numbers obtained with the most prominently used microorganisms in different cultivation methods (batch, fed-batch or continuous) are presented. The second part focuses on the downstream section and covers the applied technologies for cell removal, recovery, purification and concentration of erythritol crystals, namely centrifugation, membrane separation, ion and preparative chromatography, crystallization and drying. The final composition of the culture broth and the preparative chromatography separation performance were identified as critical points in the production of a high-purity erythritol fraction with a minimum amount of losses. During the review, the challenges for a biotechnological production of erythritol in a circular economy context are discussed, in particular regarding the usage of sustainable resources and minimizing waste streams.

**Key points:**

*• Substitution of sucrose by erythritol can be a step towards a healthier society*

*• Biotechnological production of erythritol should follow a circular economy concept*

*• Culture broth composition and preparative chromatography are keys for downstreaming*

*• Substrate, mother liquor and nutrients are challenges for circular economy*

## Introduction

Erythritol is a four-carbon sugar alcohol that is naturally present in lichen, algae, fruits, fungi and fermented food (Kasumi 1995). Due to its physicochemical, functional and nutritional properties, erythritol has been gaining importance in different manufacturing industries such as food, pharmaceutical or cosmetics (Kasumi 1995; Hao et al. 2005; BeMiller 2018). Erythritol has approximately 70–80% sweetness of sucrose. Importantly, its consumption does not affect insulin or glucose level; thus, it is considered as a safe sugar substitute for diabetic diets (Boesten et al. 2015; Martău et al. 2020). Once erythritol is consumed, it is quickly absorbed by the small intestine, distributed throughout the body and finally, up to 90% is excreted by the urine without changes. Compared to other sweeteners as xylitol and fructose, erythritol provides a higher digestive tolerance (Kasumi 1995; Boesten et al. 2015; BeMiller 2018; Rakicka-Pustułka et al. 2020).

Erythritol can be produced by chemical synthesis or biotechnological approaches. The chemical synthesis includes the catalytic transformation (e.g. Raney-nickel) of organic acids and sugars (e.g. L-tartaric acid and dialdehyde starch) (Feitosa et al. 2018), and reactions require high pressure (4-20 MPa) and temperature (120-200 °C). The chemical synthesis leads to by-products such as threitol and ethylene-glycol (Rzechonek et al. 2018). The low yields, the high complexity and the harsh operational conditions make the chemical synthesis an unfavourable option. The biotechnological production uses aerobic microorganisms such as *Moliniella pollinis, Trichosporonoides megachiliensis* and *Yarrowia lipolytica*, which require glucose as the primary carbon source (Rzechonek et al. 2018). For the erythritol market, transactions of 70,400 tons equivalent to US$198.3 million were reported in 2019, and this is predicted to increase to up to US$310 million in 2026 (Ahuja and Rawat 2020). The increase in the demand is promoted by the retail powder market and other industrial applications as for beverages, personal care and pharmaceuticals (Ahuja and Rawat 2020). The current leading companies producing erythritol are Cargill, Jungbunzlauer Suisse AG, Shandong Sanyuan Biotechnology, Foodchem International Corporation, Zibo ZhongShi GeRui Biotech, Baolingbao Biology, Mitsubishi Kagaku Food Corporation, Nikken Chemical and Frutaste (Ahuja and Rawat 2020). The raw materials, mainly represented by commercial glucose, contribute to approximately 75% of the total erythritol production costs (Ahuja and Rawat 2020).

The increasing market for erythritol on the one hand and a generally earth-wide growing demand for raw materials on the other hand reinforce the urgency to find alternative ways to cover the need for sugar substitutes like erythritol. Circular economy approaches claim design or re-design of production processes guided by the current concerns about resource depletion, waste production and management (Blomsma and Tennant 2020; Cainelli et al. 2020). To tackle the old ‘taking-transforming-using-disposing’ paradigm, a transition towards circular economy demands closed-loop processes. In 2015, the European Commission agreed on the main guidelines for circular economy, namely ‘Closing the Loop—An EU action plan for the Circular Economy’ (European Union 2015). This plan includes the sections ‘From waste to resources: boosting the market for secondary raw materials and water reuse’ and ‘Biomass and bio-based products’, in which the efficiency based on environmental eco-efficiency models play the determinant role (Toop et al. 2017; Bimpizas-Pinis et al. 2021).

Concerning erythritol production, research strategies have been implemented such as the use of alternative carbon sources, a further development of production strains, the identification of alternative erythritol-producing microorganisms or the minimization of by-product formation through metabolic engineering (Koh et al. 2003; Jeya et al. 2009; Savergave et al. 2011; Ghezelbash et al. 2014; Guo et al. 2016; Regnat et al. 2018; Liu et al. 2019; Liu et al. 2019; Martău et al. 2020; Hijosa-Valsero et al. 2021). Despite the progress reported in many studies on the upstream and cultivation stage, there is limited information about optimization strategies of the downstream part. This would be of particular importance considering that the downstream part directly affects the overall performance of the process, the waste generation, the final process yields and the overall production costs. Taken the circular economy idea into account, the design of a downstream strategy needs to identify the arrangement of unit operations that lead to a robust, safe and economically viable manufacturing process with an acceptable recovery-purity balance, a minimum waste production and a low energy consumption (Łacki et al. 2018).

This review discusses the relation between the upstream and cultivation stage and the downstream configuration and performance. Further, the main downstream stages for the biotechnological production of erythritol, i.e. it covers the main unit operations, their sequential arrangement and the challenges resulting from these configurations. As a contribution to the circular economy context, the main open loops and waste sources were identified in the separate process stages and suggestions for the implementation of non-conventional feedstocks, the treatment of residual mother liquor from crystallization, recovery of nutrients and biomass utilization are provided.

## Factors associated with the cultivation stage

The composition of the final culture broth obtained from the bioreactor defines and constrains the arrangement and performance of the downstream stage. Factors such as the carbon source and the type of culture method (batch, fed-batch or continuous) have a direct influence on the downstream process. Other relevant factors affecting the metabolism of the erythritol-producing microorganisms, such as the culture medium composition, the carbon to nitrogen ratio and the osmotic pressure influence the composition of the final culture broth. The composition is directly related to the nature and concentration level of the substances to be separated and determines the technology selection and arrangement (Hernández-Pérez et al. 2019; Martău et al. 2020). Therefore, the first part of this review describes the factors associated to the cultivation stage and their impact on erythritol yields.

### Carbon source

Several studies have assessed different carbon sources for their impact on erythritol to by-product formation by commonly used erythritol-producing microorganisms. This review distinguishes between the usage of purified, commercially available carbon sources such as glucose, xylose (Sasman et al. 2007; Guo et al. 2016) or glycerol (Rzechonek et al. 2018; Rakicka-Pustułka et al. 2020) and non-conventional substrates, which provide next to a carbon source other nutrients, such as lignocellulosic hydrolysates (Guo et al. 2016), molasses (Mirończuk et al. 2015), crude glycerol (Mirończuk et al. 2014) or residual grape (Hijosa-Valsero et al. 2021). Obtained erythritol yields and by-product formation as well as the process parameters are given for the most commonly used production organisms in Table 1 (for the conventional carbon sources) and Table 2 (for the non-conventional substrates). The by-product fraction includes for both types of substrates, mannitol, arabinitol, glycerol, ethanol and organic acids (citric acid, butyric acid and α-ketoglutaric acid) (Ryu et al. 2000; de Troostembergh et al. 2002; Rakicka et al. 2016).

**Table 1:** Overview on biotechnological erythritol production methods using conventional carbon sources

Substrate	Microorganism	Type of culture	Substrate		Erythritol production			Biomass concentration	By-products		By-product to erythritol ratio %	Cultivation medium		Ref.
			Initial	Final	g/L^b^	g/L.h	g/g^c^	g/L^b^	Compound	g/L^b^		Main components of the cultivation medium	Concentration range	
			g/L^b^	g/L^b^									g/L^b^	
Commercial glucose	*Candida magnoliae*	B	237	0	60.3	0.37	0.25	31.73	Mannitol	3.3	5.7	YE	0.23–9.2	Savergave et al. (2011)
												KH_2_PO_4_		
									Glycerol	0.3		MgSO_4_·7H_2_O		
		B	200	N.R.	20.3	0.12	0.1	N.R.	Glycerol	2.4	10.4	YE,	0.25–10	Ghezelbash et al. (2014)
												KH_2_PO_4_		
												MgSO_4_		
		FB	250	N.R.	200	1.2	0.43	±60	N.R.	N.R.	–	Peptone	2.0–20	Koh et al. (2003)
												YE		
												Phytic acid		
		FB	250	0	87.8	0.61	0.35	38.4	No by-products	–	–	YE	0.23–9.2	Savergave et al. (2011)
												KH_2_PO_4_		
												MgSO_4_·7H_2_O		
		FB	400	N.R.	164	2.8	0.41	75	Glycerol	137^a^	83.5	YE	212	Ryu et al. (2000)
									Citric acid					
									Butyric acid					
	*Candida sorbosivorans*	B	160	N.R.	60.2	0.5	0.38	N.R.	N.R.	N.R.	–	YE,	0.35–12	Saran et al. (2015)
												KH_2_PO_4_		
												MgSO_4_·7H_2_O		
												FeSO_4_·7H_2_O		
	*Pseudozyma tsukubaensis*	FB	400	0	241	2.84	0.6	22.8	No by-products	–	–	MnSO_4_·4H_2_O	2.0–15	Jeya et al. (2009)
												CuSO_4_·5H_2_O		
												Corn steep powder		
	*Trichosporonoides oedocephalis*	B	200	0.5*	50.54	0.39	0.25	27.43	Glycerol	6.4	11.3	YE	0.5–10	Li et al. (2017)
												KH_2_PO_4_		
												MgSO_4_,		
												KCl		
		B	300	5	59.34	0.49	0.2	35.1	Glycerol	30.9	34.2	YE	0.5–10	Kang et al. (2019)
												KH_2_PO_4_		
												MgSO_4_,		
												NaCl		
												CuSO_4_·5H_2_O		
												Betaine		
Commercial glycerol	*Yarrowia lipolytica*	C	300	0	199	0.8	0.66	20.6	Arabinitol	2.5	3.4	YE	0.22–26.5	Rakicka et al. (2017)
									Mannitol	2.1		(NH_4_)_2_SO_4_		
									Citric acid	2		KH_2_PO_4_		
									α-ketoglutaric acid	0.3		MgSO_4_·7H_2_O NaCl		
Commercial xylose	*Aurebasidium pullulans*	B	120	38.6	31.75	0.22	0.26	32.28	N.R.	N.R.	–	YE	0.35–17.82	Guo et al. (2016)
												KH_2_PO_4_		
												Citric acid		

*YE* yeast extract, *N.R.* not reported

^a^Total organic acid

^b^Per litre of culture

^c^Per gram of carbon source

**Table 2:** Overview on biotechnological erythritol production methods using non-conventional substrates

Substrate	Microorganism	Type of culture	Substrate		Erythritol production			Biomass concentration	By-products		By-product to erythritol ratio %	Cultivation medium		Ref.
			Initial	Final	g/L^b^	g/L.h	g/g^c^	g/L^b^	Compound	g/L^b^		Main components of the cultivation medium	Concentration range	
			g/L^b^	g/L^b^									g/L^b^	
Corncob hydrolysate	*Aurebasidium pullulans*	B	Xylose 125.7	52.7	26.3	0.18	0.12	36.78	N.R.	N.R.	–	YE,	N.R	Guo et al. (2016)
			Arabinose 46.82	36.7								KH_2_PO_4_		
			Glucose 44.18	6.7								Citric acid		
Beet molases	*Moliniella pollinis*	B	Glucose 100	0	49.52	N.R.	0.199	N.R.	N.R.	N.R.	N.R.	YE	<5	Hijosa-Valsero et al. (2021)
			Fructose 100	0										
Rosé grape must		B	Glucose 112.5	0	96.95	N.R.	0.372	N.R.	N.R.	N.R.	N.R.	YE	<5	Hijosa-Valsero et al. (2021)
			Fructose 112.5	3.0										
Red grape must		B	Glucose 104	0	90.33	N.R.	0.375	N.R.	N.R.	N.R.	N.R.	YE	<5	Hijosa-Valsero et al. (2021)
			Fructose 104	1.8										
Sugarcane molasses		B	Glucose 150	36	86.88	N.R.	0.263	N.R.	N.R.	N.R.	N.R.	YE	<5	Hijosa-Valsero et al. (2021)
			Fructose 150	13.4										
Crude glycerol	*Monilliela megachilensis*	B	200	100	30	0.17	0.15	N.R.	N.R.	N.R.	–	YE	N.R	Kobayashi et al. (2015)
	*Yarrowia lipolytica*	Repeated FB	25020% replace	N.R.	155.5	0.3	0.56	21.7	Arabinitol	0.64	10.7	KH_2_PO_4_	0.23–26.4	Mirończuk et al. (2014)
												(NH_4_)_2_SO_4_		
									Mannitol	8.1		MgSO_4_·7H_2_O		
									α-ketoglutaric acid	4.5		YE		
									Citric acid	5.4		NaCl		
		C	200	<10	81.8	0.9	0.41	19.9	Mannitol	0.8	3.0	(NH_4_)_2_SO_4_	0.22–21.3	Rakicka et al. (2016)
												NaCl		
									Citric acid	1.6		KH_2_PO_4_		
									α-ketoglucaric acid	0.1		MgSO_4_·7H_2_O		
		C	300	20	162	0.65	0.54	15.3	Arabinitol	0.6	6.4	YE	0.22–26.5	Rakicka et al. (2017)
												(NH_4_)_2_SO_4_		
												KH_2_PO_4_		
												MgSO_4_·7H_2_O NaCl		
		FB	300	0	180.3	1.25	0.53	24.41	Mannitol	7.1	8.5	YE	0.25–4.6	Rakicka-Pustułka et al. (2020)
												KH_2_PO_4_		
									α-ketoglutaric acid	6.8		(NH_4_)_2_SO_4_		
									Arabinitol	2.8		MgSO_4_·7H_2_O		
Molasses + commercial glycerol	*Yarrowia lipolytica*	FB 2 stages	Sucrose 30	0	113.9	0.85^a^	0.57	26.8	Mannitol	19	27.2		0.22–10	Mirończuk et al. (2015)
									Arabinitol	2.7		KH_2_PO_4_		
			Glycerol 200						Citric acid	11.8		NaCl		
									α-ketoglutaric acid	2.8				

*YE* yeast extract, *N.R.* not reported

^a^Yield from glycerol

^b^Per litre of culture

^c^Per gram of carbon source,

Because of easy handling, safety, purity and selectivity towards erythritol, high-concentrated glucose syrup (16–40% (w/v)) is the most used carbon source until now both in industrial and research scale. Jeya et al. evaluated the erythritol production by the basidiomycetous yeast *Pseudozyma tsukubaensis* using different carbon sources (Jeya et al. 2009). The study included the usage of glucose, fructose, galactose, mannose, sucrose, sorbose, glycerol and lactose as carbon sources. The results led to highest carbon source assimilation and erythritol concentration using glucose as carbon source, followed by sucrose and mannose (Jeya et al. 2009). Saran et al. isolated the yeast *Candida sorbosivorans* SSE-24, which had a selective erythritol production using glucose as carbon source, in contrast to the use of glycerol, starch or sodium acetate, for which no erythritol production was observed (Saran et al. 2015).

Commercial glycerol has been identified as preferable carbon source for erythritol production using *Monilliela megachilensis* SN-G42 (Kobayashi et al. 2015) and *Yarrowia lipolytica* MK1 (Mirończuk et al. 2014; Mirończuk et al. 2015; Rakicka-Pustułka et al. 2020) obtaining higher yields and a higher erythritol to by-product ratio compared to commercial glucose. Summarizing, for the commercial carbon sources, erythritol yields of 0.1–0.6 g/g glucose, 0.26 g/g xylose and 0.66 g/g glycerol were reported (Table 1). These values are close to the maximum theoretical erythritol yields of 0.678 g/g hexoses and 0.884 g/g glycerol (Hijosa-Valsero et al. 2021). Even though glucose and glycerol provide a high selectivity for erythritol production, their high price and volatilities (approx. 480 $/ton glucose and 270-500 $/ton refined glycerol) are clear disadvantages. Aside from that, socioeconomical concerns arise about the usage of sugars or highly refined substrates for producing sugar substitutes.

The use of non-conventional substrates is considered a key point in a circular economy context (Cardona-Alzate et al. 2020). This matches well with the increasing interest in a utilization of residual biomass from agricultural or industrial activities either in stand-alone process or in biorefinery concepts. Summarizing, for non-conventional substrates, erythritol yields of 0.15–0.57 g/g crude glycerol and 0.12–0.375 g/g of other non-conventional substrates were reported (Table 2). Besides being a highly available compound, crude glycerol contains various mineral nutrients that can be beneficial for cultivation purposes. The most commonly used erythritol-producing microorganisms can grow on it and synthetize erythritol with the advantage that remnant compounds (e.g. NaOH, NaCl, Ca, K or non-glycerol organic matter) do not need to be removed before cultivation (Mirończuk et al. 2014; Kobayashi et al. 2015). Furthermore, Guo et al. assessed the erythritol production of a mutant strain of the yeast-like fungus *Aureobasidium pullulans* using corncob hydrolysate as carbon source. They detected an incomplete sugar consumption with remaining xylose, arabinose and glucose concentrations of 58.0%, 20.2% and 84.8%, respectively, and a final erythritol concentration that was only 17% lower than the control using glucose (Guo et al. 2016). The evaluation of alternative substrates for erythritol production also included the usage of molasses (Mirończuk et al. 2015) and fungal-pretreated soybean residues (Liu et al. 2017) for cultivation of *Y. lipolytica*, corncob hydrolysate for cultivation of *A. pullulans* (Guo et al. 2016), sugarcane molasses, beet molasses, rosé and red grape must for cultivation of *M. pollinis* (Hijosa-Valsero et al. 2021) and wheat straw for cultivation of *Trichoderma reesei* (Jovanović et al. 2014). Studies on emerging microorganisms for erythritol production included the latter fungus (Jovanović et al. 2014; Regnat et al. 2018). It has developed a saprotrophic lifestyle and therefore produces high amounts of enzymes, in particular cellulases and hemicellulases. Such saprobes would be interesting production organisms for erythritol from all kinds of lignocellulosic feedstocks. Jovanović et al. assessed the usage of delignified wheat straw as substrate for cultivation of a recombinant *T. reesei* strain and observed besides good-growth erythritol concentrations of only around 5 mg/L (Jovanović et al. 2014). This pointed to the need for improving the biochemical flux towards erythritol and triggering its secretion as the main challenge for using this microorganism.

While the presence of additional compounds next to the carbon source in non-conventional substrates can be advantageous, these substrates might also raise a need for pretreatment and/or detoxification. For example, Hijosa-Valsero and co-workers reported inhibition in growth and production of erythritol using beet molasses without pretreatment due to the high level of cations in the untreated feedstock. This inhibitory effect was reduced by a detoxification using a cation exchange column filled with Amberlite IR-120 resin (Hijosa-Valsero et al. (2021)). Other studies have reported on the utilization of lignocellulosic biomass to produce xylitol. A pretreatment coupled to detoxification strategies was needed to overcome the recalcitrance of lignocellulose and to remove inhibitory compounds arising from sugar solubilization (Wei et al. 2010; Kresnowati et al. 2017; Kumar et al. 2019). In case of crude glycerol, residual oils, fatty acids, inorganic compounds and proteins could cause microbial inhibition or rheological restrictions, and crude glycerol also demands control of the pH (Kobayashi et al. 2015).

### Composition of the cultivation medium

Increase in cell permeability, prevention of damage by lysis and increase in erythrose reductase activity can be reached by the addition of ion metals and surfactants and modifying the C:N ratio (Savergave et al. 2011; Kang et al. 2019; Rakicka-Pustułka et al. 2020).

The addition of metal ions like Mn^2+^, Cu^2+^ and Zn^2+^ as salts has been gaining importance in the optimization of the culture medium for strains like *Torula* sp. and *Y. lipolytica* (Rzechonek et al. 2018)*.* Some studies have discussed the positive effects of Cu^2+^ and Zn^2+^ on the increase in the erythrose reductase activity (Lee et al. 2000; Tomaszewska et al. 2014; Janek et al. 2017) or described the role of Mn^2+^ as a facilitator for erythritol transport through the cell membrane (Lee et al. 2000; Tomaszewska et al. 2014). Lee et al. (2000) reported increases of 34% and 33% in erythrose reductase activity and final erythritol concentration by the addition of 10 mg/L CuSO_4_·5H_2_O and 10 mg/L MnSO_4_·4H_2_O, respectively.

Kang et al. (2019) assessed four different surfactants (Betaine, Tween 20, Tween 80, Span 20 and Triton X-100) to determine the effect on the production of erythritol by *T. oedocephalis*. A Betaine dosage of 0.5 g/L leads to a 10% increase in the erythrose reductase activity. Besides, this experiment did not present cell disruption compared to the control culture. This result was attributed to the capability of betaine to protect the morphology and viability of the microorganism (Kang et al. 2019).

According to Mirońzuc et al. and Rakicka et al., the preferred formation of product versus biomass can be modulated by the C:N ratio (Mirończuk et al. 2015); for example, higher erythritol production instead of a high biomass synthesis can be obtained under limited nitrogen conditions (Rakicka et al. 2017). Several groups have considered nitrogen in their studies on medium optimization, including organic nitrogen sources such as yeast extract or inorganic sources as ammonium sulphate (Savergave et al. 2011; Rakicka et al. 2016; Rakicka-Pustułka et al. 2020). Savergave et al. determined an optimal yeast extract concentration of 10 g/L for the culture of *C. magnoliae*, producing a maximum erythritol concentration of 57 g/L. However, it should be noted that high nitrogen levels can lead to increased concentrations of by-products of up to 40–50% of total erythritol produced (Savergave et al. 2011). Rakicka et al. determined the capability of *Y. lipolytica* to consume glycerol as carbon source using yeast extract (9.75 g/L) and ammonium sulphate at different concentration levels (2.3 g/L and 4.6 g/L). The usage of 4.6 g/L ammonium sulphate led to erythritol yield of 0.52 g/g which is higher than the obtained yield using the low level of ammonium sulphate (0.29 g/g) or yeast extract (0.47 g/g) (Rakicka et al. 2016).

The viscosity of the culture medium represents an important factor to be considered, as an increase of viscosity reduces the oxygen transfer rate and promotes the production of other undesired by-products such as ethanol (de Troostembergh et al. 2002). The production of polysaccharides during the cultivation results in a rapid increase of the viscosity and makes it difficult to recover highly pure crystals as it reduces the crystallization rates and purity of the final product. According to de Troostembergh et al., polysaccharide concentrations in the order of 2% (wt. based on erythritol) can already limit the final erythritol purity of up to 80% wt. and results in an ethanol production in the order of 22% wt. (based on erythritol) (de Troostembergh et al. 2002).

### Cultivation conditions

Different cultivation modes including batch, fed-batch or continuous cultivation have been evaluated for erythritol production. For example, in case of *C. magnoliae*, the highest erythritol concentration (namely 200 g/L (Koh et al. 2003)) was obtained with fed-batch cultivation using commercial glucose as carbon source (Table 1). With batch cultivations using the same microorganism and carbon source, only three to ten times lower erythritol concentrations (60.3 and 20.3 g/L; Savergave et al. 2011; Ghezelbash et al. 2014) were obtained (Table 1).

For the non-conventional substrates, the cultivation of *Y. lipolytica* using crude glycerol in fed-batch mode gave the highest erythritol concentration (180.3 g/L; Rakicka et al. 2017) followed by the same organism on the same substrate in continuous cultivation mode (162 g/L; Rakicka-Pustułka et al. 2020) (Table 2).

Productivity and yields can be also linked to the osmotic pressure. The osmotic pressure influences cell growth and cell osmolarity on the one hand, and on the other hand, high levels of osmotic pressure can induce the activity of erythrose reductase, hence promoting the production of erythritol (Rakicka-Pustułka et al. 2020). Besides, a higher osmotic pressure promotes the excretion of products such as glycerol and erythritol, protecting the cell against plasmolysis (Li et al. 2017).

The osmotic pressure can be adjusted through the carbon source and/or the salt concentration. Yang and co-workers reported an increase in the production of erythritol caused by the use of sodium chloride as osmotic agent in contrast to the utilization of glycerol. They found an increase in the expression of 26 different proteins, some of them involved in erythritol synthesis (Yang et al. 2015). Liu et al. reported increased excretion of glycerol by *T. oedocephalis* as a mechanism for osmotic pressure stabilization in response to potassium chloride concentrations applied above the optimum (Li et al. 2017). For batch cultivations, the initial concentration of the carbon source is about 120–300 g/L, which is mainly used for the growth of the microorganisms, while the osmotic pressure usually is adjusted by the addition of salts once the microorganisms reached a specific biomass concentration (Li et al. 2017).

On the other hand, the fed-batch cultivation mode itself is a strategy for osmotic pressure modulation. This modulation is achieved through an initial carbon source concentration of 250–400 g/L (corresponding to low osmotic pressure), which is a suitable condition for the growth of the microorganism. Then, feeding a higher amount of carbon source increases the osmotic pressure as well as the erythritol production rate (Kim et al. 1997). Due to the advantages of the fed-batch culture mode, different approaches have been performed to evaluate the modulation of the osmotic pressure during the cultivation. These approaches include two-stages fed-batch (Mirończuk et al. 2015) and repeated fed-batch strategies (Mirończuk et al. 2014).

Mirończuk et al. assessed the two-stage fed-batch approach using two different carbon sources at different concentrations (molasses 30 g/L and commercial glycerol 200 g/L) for the biomass growth stage and the erythritol production stage, respectively (Mirończuk et al. 2015). The authors reported an increase in the osmotic pressure of 8.3 times to 11.6 times (2.5–2.8 osmol/kg) during the second stage (Mirończuk et al. 2015). Although this approach led to a high erythritol yield (0.57 g per g carbon source), the concentration of erythritol (113.9 g/L) and the by-product to erythritol ratio (0.272) were still lower compared to other fed-batch configurations.

Mirończuk et al. also assessed the repeated fed-batch strategy using residual glycerol as carbon source by replacing 20% of the culture broth by fresh medium. The complete cycle lasted 64 days with 11 cultivation medium replacements. This approach showed a similar erythritol yield (0.56 g per g carbon source), higher concentration of erythritol (155.5 g/L) and a lower by-product to erythritol ratio (0.107) compared to the two-stage fed-batch approach. The authors concluded the overall performance was affected by the type of carbon source and the amount of medium replaced (Mirończuk et al. 2014).

Considering alternative substrates and cultivation modes, solid-state fermentation (SSF) was evaluated for erythritol production with the mutant strain *Y. lipolytica* M53-S using oil crop residues supplemented with waste cooking oil and sesame meal. The obtained total erythritol concentration was 185.4 mg/g of dry substance (Liu et al. 2019). Liu et al. also assessed the production of erythritol using biochars from different residues (rice husk, wheat straw, mushroom and pig manure) as an enhancing agent of the carbon source consumption in SSF with the mutant strain *Y. lipolytica* M53-S. The obtained erythritol concentration was 222.5 mg/g of dry substance (Liu et al. 2020). The main advantages offered by SSF include more manageable operation and the low consumption of energy and water. However, this mode of cultivation is still a matter of research due to the low yields (similar to batch cultures), heterogeneous mass transfer, troubles for oxygen distribution and longer times of cultivation required (Liu et al. 2017; Liu et al. 2019; Liu et al. 2020).

### Conclusions from the section

Glucose as the mainly conventional carbon source used gives higher productivities, yields and final concentrations of erythritol than non-conventional substrates. Notwithstanding, recent studies have shown improvements in final erythritol concentrations using diverse non-conventional substrates like lignocellulosic feedstock, crude glycerol, agro-industrial or dairy residues. Such substrates have the advantage of high availability and the provision of carbon sources. With regard to the demand for macro- and micro-nutrients in cultivation processes, their utilization offers a natural source of ions, vitamins or nitrogen, thereby decreasing or minimizing the demand of salts (Tomaszewska et al. 2014; Kobayashi et al. 2015; Fayet et al. 2018; Hausjell et al. 2019). Further, the production of erythritol using such substrates can be designed as a biorefinery approach making an integral utilization of the substrates to obtain different families of products (Serna-Loaiza et al. 2019; Awasthi et al. 2020; Sebastián-Nicolás et al. 2020). Considering these points, more research towards erythritol production in a circular economy context would be needed.

In terms of the cultivation mode, the fed-batch configuration showed higher yields and concentrations by offering the possibility of controlling osmotic pressure through the additional carbon source with or without medium supplements or osmotic agents to the culture broth.

### Downstream processes for the biotechnological production of erythritol

**Fig. 1:**
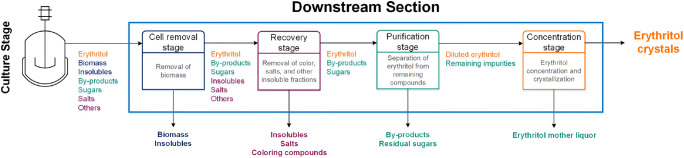
Scheme of the downstream process for erythritol production

Figure 1 presents a schematic overview on the stages of an erythritol production process starting from the cultivation followed by the major stages of the downstream section, which are (i) the cell removal, (ii) the recovery of erythritol (including the separation of salts and insoluble compounds), (iii) the purification and (iv) the concentration. Each stage and the corresponding, available technologies will be discussed in the following sections separately.

### Cell removal stage

The cellular biomass plays an important role in biotechnological erythritol production as the biofactory and an important by-product at the same time, reaching concentrations of about 15.30 to 75 g/L in the culture broth (Tables 1 and 2). Since erythritol is an extracellular product, the downstream strategy needs to allow the removal of non-soluble by-products and remaining microorganisms. Both can affect and interfere with yields of further operations by clogging membranes or resin columns, as well as with the performance of heat exchange devices (Morioka et al. 2000). Different approaches for the biomass disposal include recirculation (Morioka et al. 2000), cell inactivation (Sasman et al. 2007) and valorization according to the physicochemical characterization (e.g. protein and lipid fraction) (Ekpeni et al. 2014; Mirończuk et al. 2014; Patrignani et al. 2020). Importantly, the type of cultivation mode implicates the possibility to separate or even reuse biomass.

The main focus for batch cultivations is to inactivate and separate the biomass, which is performed by heating the culture broth with temperatures up to 70 °C (Sasman et al. 2007). Several studies recommend processes at temperatures close to the inactivation point (50–90 °C) avoiding biomass re-growth or microbiological contamination (Maeda et al. 1995; Morioka et al. 2000; Sasman et al. 2007). Then, the solid fraction is separated from the liquid fraction by centrifugation (Toshihiro et al. 1993; Maeda et al. 1995), vacuum filtering (pre-coated rotary filter) (de Troostembergh et al. 2002) or membrane filtration with pore sizes not bigger than 0.1 μm and a molecular weight cut-off not lower than 10,000 Da (Maeda et al. 1995; Sasman et al. 2007). Ultrafiltration performed close to the isoelectric point (pH 3.5-5.5) can facilitate the precipitation and removal of enzymes and protein fractions, reducing the foaming as well as interferences in ion exchange operations (Morioka et al. 2000; Kresnowati et al. 2019).

Regarding continuous cultivation, the aim is not only the inactivation of the biomass, already described in the previous paragraph, but also the recirculation and reuse of the biomass. Maeda et al. assessed a microfiltration or ultrafiltration flat membrane coupled to the bioreactor for cell separation to obtain an erythritol-enriched permeate fraction and a retentate fraction (rich in biomass slurry). The microfiltration membranes should have a maximum pore size of 1 μm, and the ultrafiltration membranes should have a maximum cut-off molecular weight value of 10,000 Da (Maeda et al. 1995). The cultivation was performed in continuous mode, and once the concentration of glucose decreased to 5%, the authors started filtrating at a constant transmembrane pressure of 0.98 bar. The retentate fraction was then recirculated, ensuring a cellular concentration around 100 g/L (dry basis). Using this approach, the authors reported a 2.67-fold increase of the erythritol productivity (Maeda et al. 1995).

### Recovery stage

This stage aims to remove insoluble fractions, ionizable compounds and colour from the biomass-free culture broth and to isolate the erythritol fraction in a clarified solution that can be processed in the purification step. For these purposes, different unit operations as ultrafiltration, ion exchange and concentration can be used. The effluent of the separation stage is a stream mainly composed of residual carbon source, erythritol and main by-products such as glycerol and other polyols.

### Ultra- and nanofiltration

Depending on the approach used for the removal of the biomass, enzymes and insoluble substances could remain in the culture broth, e.g. after the centrifugation. Such impurities could cause interference with heat exchangers, lead to foaming, clog ion exchange units, foul membranes or increase resistance to mass transfer (Morioka et al. 2000; Poletto et al. 2015).

After heating at 70°C, which ensures inactivation of the microorganisms and thermal precipitation of the enzyme and protein fraction in the culture broth, Rakicka et al. used a membrane filter, namely Cell-Free type Sampling (Bioengineering) with a flat membrane made of polysulfone (diameter of 45 mm, pore size 0.45 mm) for the separation of biomass (Rakicka et al. 2016). Ultrafiltration is also used in downstream and upstream stages to produce other polyols like xylitol (Kresnowati et al. 2017; Kumar et al. 2019) Kresnowati et al. reported the removal of biomass and protein fractions from the culture broth containing xylitol, which improved the efficiency of further operations as electrodialysis (Kresnowati et al. 2019). For improved erythritol purification, Li et al. (2020) and Zhao et al. (2020) included nanofiltration after the microfiltration as an additional step in order to remove remaining macromolecules before the subsequent ion exchange.

#### Ion exchange for desalting

The biomass-free fraction can be submitted to ion exchange treatment to remove ionizable compounds, such as inorganic salts or organic acids (e.g. acetic acid), and/or colouring compounds from the cultivation broth. Besides the addition of inorganic salts to the cultivation medium, hardness ions such as calcium and magnesium could be introduced by the utilization of tap water or certain substrates (e.g. glycerol or hydrolysates) (Jovanović et al. 2014; Rakicka et al. 2017). A softening step to remove the hardness from the culture broth can be performed using sodium-based strongly acidic cation exchange resins and weakly acidic cation exchange resins (Morioka et al. 2000). For a good separation efficiency of further chromatographic separation steps, some studies have suggested to perform such a softening process before the chromatographic separation (Sasman et al. 2007) or coupled to the desalination process (Rakicka et al. 2016).

Rakicka et al. reported a desalination process using a four-column arrangement including ion exclusion and ion exchange separation (Rakicka et al. 2016). The ion exclusion stage was performed using a sodium monospheric cation exchange column (Lewatit S3783-Bayer) and deionized water (20 °C) as eluent at a flow rate of 1 m^3^/m^3^*h. They obtained three main phases: a saline waste (0.22 Bed Volume (BV)), a transition fraction free of products (0.22 BV) and a desalinated-rich erythritol fraction (0.25 BV). During the ionic exchange treatment, the desalted stream was passed through three ionic-exchange beds: a strong cationic-exchange bed (H^+^, Lewatit S112-Bayer), a weak anionic-exchange bed (OH^-^, Lewatit MP64-Bayer) and a mixed bed composed of strong cationic and weak anionic resins. The overall arrangement allowed a sodium chloride removal and an osmotic pressure reduction of 99.3% and 76.2%, respectively (Rakicka et al. 2016).

Sasman et al. reported an approach for the removal of salts after softening (i.e. removal of calcium and magnesium ions) and chromatographic separation of erythritol. The salts fraction was removed at 50°C using cationic (Purolite C-155S) and anionic (Purolite A103S) resins exchanging hydrogen and hydroxyl ions. An additional salt removal stage was performed by passing the stream through a mixed bed composed of a strong anion and strong cation exchange resins (Purolite C155S and A51MBS resins) (Sasman et al. 2007).

The regeneration process is regular maintenance step for exhausted ion exchange resins. It demands the use of brines or other regenerants (NaCl, NH_4_, NH_4_HCO_3_, Na_2_CO_3_, HCl, H_2_SO_4_, among others) to remove the ions attached to the resins, usually salts and other components from the culture broth (Chandrasekara and Pashley 2015), and replacing them with the original exchangeable ion. This procedure involves multiple steps, namely backwash, chemical injection of regenerant solutions, slow rinse and fast rinse, leading to a water demand—and finally wastewater generation—equivalent to 7 times the resin volumes (Purolite 2021). The amount of waste generated and the extensive utilization of regenerants should point to the need to develop technological solutions that improve the ecological efficiency of this step.

#### Primary concentration

After removing ionizable compounds and small molecules, the remaining fraction can be submitted to an initial concentration stage by evaporation to remove water and volatile compounds (e.g. acetoin) and to improve the efficiency of the chromatography separation by increasing the solid content up to 35–50% (Maeda et al. 1995), (Toshihiro et al. 1993). Among the different evaporators, falling film evaporators offer a controlled heat transfer and prevent or reduce the foaming effect (Morioka et al. 2000). To ensure adequate and stable conditions, the optimal pressure operation window (lower and upper pressures) needs to be determined. The lower pressure is limited to a value at which no foaming is observed. This reduces the risk of microbiological contamination, in particular when the concentration step is conducted at temperatures used for microbiological growth. The upper pressure limit is the value at which neither polymerization of polyols occur (Liu et al. 2010) nor Maillard reactions which are interactions between the carbon source and amino acids at high temperatures and longer heating times (Morioka et al. 2000). These reactions produce components that could introduce colour, flavour or aroma to the final product (Labuza et al. 1998).

Several authors reported the use of vacuum evaporation for the increase of solids content (Morioka et al. 2000; Sasman et al. 2007; Rakicka et al. 2016). Morioka et al. reached 40 %wt. solid concentration by using a quadruple-effect shell and tube falling film evaporator at pressures and temperatures between 1.43 and 3.87 psi and 46 and 70°C, respectively. No foaming was observed during the operation, which reduced erythritol losses (Morioka et al. 2000).

### Purification stage

This stage aims to separate the erythritol fraction from the residual carbon source and main by-products as glycerol and other polyols. The separation principle that should govern this stage is the interaction between partially charged sites on the surface of the molecules and different matrix as chromatographic resins and activated charcoal. Ion chromatography and decolouration operation units can be used to obtain a stream with a high concentration of erythritol and a low concentration of impurities.

### Preparative chromatography

The main goal of preparative liquid chromatography is to separate and isolate compounds from simple or complex mixtures (Waters Chromatography Division of Millipore 1987). Rukowicz et al. (2020) studied the separation efficiency of the erythritol-sodium chloride mixture. Six column overload levels (10–60% BV) were assessed in a chromatographic column (100 cm length, 26 mm internal diameter) using a monospheric strongly acidic cation exchange resin Lewatit S1567 (Styrene-DVB, Na^+^ form, 0.6 mm diameter). A reduction in the separation efficiency with the increase of volume overload was detected. However, the erythritol dilution obtained at low overload levels can pose an issue for further concentration stages. The better separation efficiency was found at 30–40 % column load with separation efficiencies between 42 and 58%.

Water is the most used eluent for neutral or weakly ionized compounds as erythritol and erythritol by-products (Haddad and Jackson 1990). Paanamen et al. recommend that the feed and the eluent are preheated at temperatures of around 65–95°C facilitating the separation performance and an alkaline pH (6–11) (Paananen et al. 2002). They assessed a preparative chromatographic separation using a 70-cm length and 4.5-cm diameter column packed with a weakly acidic ethyl acrylate 6% DVB cross-linked resin with Na^+^, used for the separation of sodium chloride, betaine, erythritol, glycerol, inositol, sucrose, mannitol and α-amino acid. Samples were fed at 80 °C and 4 mL/min using water as eluent. According to the results, one chromatography column was insufficient to separate erythritol, mannitol and glycerol, which had almost the same elution time (Paananen et al. 2002).

Sasman et al. assessed a batch preparative chromatography for erythritol recovery using a cationic acid resin (Purolite PCR-821). The obtained erythritol-rich fraction was submitted to the desalination process described in the corresponding section above (Sasman et al. 2007).

Morioka et al. (2000) performed the preparative chromatography separation by using a simulated moving bed system consisting of a sequential arrangement of four columns filled as follows: (i) strongly acidic, cationic DVB polystyrene sulfonic acid cross-linked resin (DIAION UBK-550, Mitsubishi Chemical Corp, Na^+^ type); (ii) a strongly acidic, cationic resin (DIAION SK1B- Mitsubishi Chemical Corp, H^+^ type); (iii) a weakly basic, anion resin (DIAION WA30- Mitsubishi Chemical Corp, OH^-^ type) and finally, (iv) a mixed bed of the before mentioned resins (DIAION PA408, Mitsubishi Chemical Corp). The fed fraction was composed of a mixture of the softened culture broth and the saturated mother liquor from the crystallization step (Morioka et al. 2000). The purpose of the first column was to perform a separation into two eluting groups: the first group contained salts, colour compounds, high-molecular–weight compounds and polysaccharides and the second group contained the erythritol fraction that coelutes with glycerol. Then, erythritol and glycerol are separated by the second to the fourth column obtaining a stream with an erythritol fraction of around 3–30 %wt. (Morioka et al. 2000).

#### Decolouration

Decolouration removes pigments and other residual compounds from the culture broth, improving the crystallization yield and erythritol purity. It is also widely used for other polyols, such as xylitol (Martínez et al. 2007; Misra et al. 2011; Martínez et al. 2015).

The location of the decolouration step in the downstream section is still a matter of discussion. The two mostly used approaches include this step either in the recovery or in the purification stage. Li et al. assessed the decolouration with activated carbon of micro- and ultrafiltered, biomass-free culture broth early in the recovery stage (Li et al. 2020). The main benefit of an early elimination of odours, colouring macromolecules, is the improved efficiency of the preparative liquid chromatography. On the other hand, Morioka et al. assessed the decolouration stage after the preparative liquid chromatography to remove contaminants capable of affecting the final quality of crystals. However, both groups identified the dosage, temperature, time of contact and stirring velocity as the key parameters. Suitable operational conditions can be determined by colour measurement or analysing the recovery of the erythritol and crystallization yield (Toshihiro et al. 1993; Morioka et al. 2000).

Another group reported a dosage of activated carbon equivalent to 1–1.5 % of the culture broth in a filtration column including diatomaceous earth, activated carbon, cardboard and paper filter as a key step for removing impurities and ensuring better performance of further operations (Wang et al. 2016).

### Concentration stage

This stage aims to obtain erythritol crystals from the purified stream containing erythritol and residual impurities. Secondary evaporation, crystallization, crystals separation, washing, drying and sieving are the steps necessary to reach final, crystallized erythritol. Due to the moderate erythritol solubility, crystallization is the crucial operation to be developed during this downstream stage (Fujii et al. 2015). Hence, it is advantageous to start with a liquid fraction as pure as possible (Martínez et al. 2007; Eroma et al. 2010; Saran et al. 2015). For example, Saran et al. reported a reduced erythritol recovery yield of 52.24% due to residual by-products present in the supersaturated solution (Saran et al. 2015).

#### Secondary concentration

Similar to the primary concentration, the purpose of this step is to evaporate part of the water in the solution. Morioka et al. reached 48%wt. solid concentration using the same conditions as for the primary concentration treatment: a quadruple-effect shell and tube falling film evaporator at pressures and temperatures between 1.43 and 3.87 psi and 46 and 70°C, respectively (Morioka et al. 2000). Toshihiro et al. assessed the influence of the erythritol concentration in the liquor before crystallization on the mechanical properties of the final crystals. Concentrations in the liquor higher than 67%wt produce breakable, clump-forming and cake-forming crystals. To improve the mechanical properties of the crystals, it is recommended to reach concentrations between 40 and 55%wt of erythritol in the stream obtained from the evaporation stage (Toshihiro et al. 1993; Morioka et al. 2000).

#### Crystallization

Erythritol has a negative heat of crystallization of −108 kJ/kg. The energy released demands thermal controlling to obtain a stable, efficient crystallization process. At supersaturation conditions, the solution passes through two phases: nucleation and crystal growth. The nucleation phase is the formation of erythritol clusters made of gathered molecules. Clusters are transformed into a crystal nucleus after reaching a critical size. The supersaturation level and temperature determine the critical size of the initial nuclei clusters. Once the critical size is exceeded, the crystal growth stage starts. This stage occurs simultaneously with the clustering process until the equilibrium between diluted and crystalline erythritol is reached (Tyapkova et al. 2012). Tyapkova et al. assessed factors influencing the crystallization of erythritol, for example the effect of temperature over the saturation, the temperature of saturated erythritol solutions, the time of the storage of saturated erythritol solutions and the cooling rate. Slower cooling rates promote the formation of larger, resistant and compact crystals (average length 200–400 μm) (Tyapkova et al. 2012). Erythritol solubility increases as temperature increases. Values of 33, 54 and 257 g of erythritol per 100 g of water are obtained at 5°C, 20°C and 80°C, respectively (Tyapkova et al. 2012). Typical process temperatures in the order of 70–80°C ensure a high concentration of erythritol in the liquid phase and increase the recovery of erythritol.

High content of impurities in the crystals increases the hygroscopicity, leading to unstable management and storage (Morioka et al. 2000; Eroma et al. 2010). Morioka et al. obtained erythritol crystals with 99.9% purity using a cooling rate of 7.5 °C/h from 70 to 15 °C. For promoting the crystal growth stage, a crystal seed of 0.01%wt based on the erythritol concentration was added at 42 °C (3 °C below the saturation temperature) (Morioka et al. 2000).

Toshihiro et al. performed the crystallization of erythritol from an initial temperature of 60 to 20 °C with a constant cooling rate of 5°C/h. The formed crystals were washed with water using half of the weight of the wet crystals. The residual mother liquor contained 26.3%wt. of erythritol and a reduced amount of acetoin as a by-product by order of 10.5 ppm (Toshihiro et al. 1993).

Alternative technologies to conventional crystallization have been developed and patented within the last 10 years using aids to ensure high uniformity in particle size and fluidity which reduces energy consumption during concentration stages (lower concentration requirements) and waste generation. This can be reached by addition of alcohol (usually ethanol, propanol, isopropanol or methanol) as an antisolvent leaching agent by up to 75%. Antisolvents are used in crystallization processes to reach saturation or supersaturation of solution in shorter times and affects the nucleation and crystal growth processes (El Bazi et al. 2017). The addition of the antisolvent to a 45–50% wt. solid mother liquor was performed to control the particle size of the crystals. The final particle size was adjusted by varying the amount and particle size of the seed. Crystals with 40–80 mesh were obtained after adding 1–3 %wt. seed (170–200 mesh of particle size). Finer 30–60-mesh crystals were obtained by adding a lower amount, namely 0.5–1.5 %wt. of the seed (120–150 mesh of particle size) (Jiang et al. 2013). The authors claimed a reduction in the total crystallization time to less than a third of the conventional crystallization using ethanol as aid compared to without aid and still obtaining 80% recovery and 99.5% crystal purity (Jiang et al. 2013).

The approach proposed by Zhang et al. (2017) consists of a continuous oscillating flow film crystallization arrangement developed in two steps. The first step was performed in a hollow membrane module where the mother liquor is fed at 40–80 °C to the tube side. The water was removed by passing through the membrane to the shell side, which increases the saturation level of the mother liquor leaving the membrane. However, due to concentration differences and crystal deposition on the membrane surface, concentration polarization and gel polarization phenomena can occur, which affect the flux through the membrane and the overall efficiency. To overcome this drawback, an oscillation of 1–10 Hz of frequency and 1–5 cm of amplitude is applied while the distribution of this oscillation is ensured by baffles (rod along, circular installed at intervals) installed in an axial direction to the flow. The supersaturated mother liquor was sent to a fluidized crystallizer with internal recirculation while the crystals formed are removed from the bottom of the crystallizer. This approach yielded uniform crystals without seeding.

Zhang et al. (2018) patented a continuous process for erythritol crystallization using a membrane crystallization module. This module was composed of a hollow tubular membrane module capable of performing preferential pervaporation of antisolvent (ethanol, propanol or isopropanol 25–40 °Gay-Lussac) (Wang et al. 2017a). The experimental setting sent the diluted mother liquor (30–45 % wt.) to the tube side of the hollow membrane. Besides, the antisolvent was sent to the shell side, with a recommended antisolvent-to-solvent flow ratio of 0.5–5 times ethanolic eluent addition to the mother liquor. This promotes local high supersaturation phenomena a large and uniform average particle size for the erythritol crystals. Then, the erythritol crystals leave the hollow tubular membrane module as a slurry to be separated from the liquid fraction. The liquid fraction is submitted to evaporation, recovering the antisolvent and recycling the mother liquor to the system.

#### Crystal separation

After the formation of the crystals, the next step consists of their separation from the liquid solution. Centrifugal force can be applied for the separation of erythritol crystals from the saturated mother liquor. It needs to be considered that lower centrifugal forces implicate large amounts of remaining mother liquor in the crystals, whereas high centrifugal forces can fracture the crystals (Jiang et al. 2013; Wang et al. 2016). Morioka *et* al. suggested values around 100 to 300 g to separate the crystals avoiding fractures (Morioka et al. 2000). Further, Wang *et* al. emphasized in the importance of washing using a vibrating fluidized bed to protect the crystal shape and size particle. After centrifugal separation, removing the excess of the mother liquor, which remains around the crystals, is necessary. This separation can be performed by spray-washing the crystals with cold water (10–20°C) in a preferred ratio of 0.2–0.5 (%wt.) to avoid erythritol losses due to dilution effects (Morioka et al. 2000).

#### Drying

After having the washed crystals, the final step is the removal of the water present on the surface of the crystalline structures. Erythritol can be dried as common sugars, using technologies such as a rotary drum dryer (co-current and counter-current airflow) (Maeda et al. 1995) or a fluidized bed dryer (Morioka et al. 2000). Because of the large solid-gas contact surface and the mixing degree, the mass transfer rates are favoured by using fluidized bed dryers (Touil 2017). The main parameters to be considered by this technology are the gas temperature, pressure, flow and particle size (Ambrosio and Taranto 2002; Touil 2017).

### Conclusions from the section

A downstream approach for erythritol production must offer an arrangement of the unit operations that is capable of removing biomass, other macromolecules, salts, odour- and colour-causing compounds and by-products to obtain high recovery yields and purity.

De Troostembergh et al. patented a downstream approach for a cultivation using Moniliella tomentosa *pollinis* TCV364. They claimed that it is free of extensive refining processes and gives erythritol crystals with 98–99% w/w purity. The approach consists of biomass removal by filtration, followed by vacuum evaporation obtaining solids concentration higher than 80%, a cooling crystallization and centrifugal washing that leads to a total erythritol recovery of 85% (de Troostembergh et al. 2002). However, their recovery yields are limited by the generation of by-products during the culture stage (i.e. polysaccharides and ethanol). Several other research groups have proposed different downstream configurations based on size, affinity and volatility separation principles. According to reports in literature and as outlined above, they included membrane separation, evaporation, decolourization, ion resin exchange, preparative chromatography, crystallization and sieving technologies (Toshihiro et al. 1993; Morioka et al. 2000; Sasman et al. 2007; Rakicka et al. 2016; Li et al. 2020; Zhao et al. 2020).

**Fig. 2:**
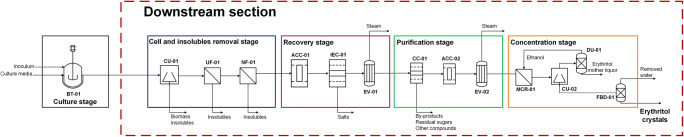
Proposed downstream configuration. BT-01 bioreactor, CU-01 centrifuge, UF-01 ultrafiltration unit, NF-01 nanofiltration unit, ACC-01 activated charcoal column, IEC-01 ion exchange column, EV-01 evaporation unit, CC-01 chromatographic column, ACC-02 activated charcoal column, EV-02 evaporation unit, MCR-01 membrane crystallization unit, CU-02 centrifuge, DU-01 distillation unit, FBD-01 fluidized bed dryer

Figure 2 presents a scheme, which shows in the authors’ opinion the optimal downstream configuration considering the results of the available studies that analysed the different unit operations.

The 1st stage needs to remove biomass and macromolecules (e.g. enzymes or polysaccharides) by centrifugation, ultrafiltration and nanofiltration to avoid any interferences by deposition or unwanted interaction in subsequent operation units.

The 2nd stage should comprise decolouration, desalting ion exchange and primary evaporation. The decolouration process was introduced as the first unit operation of because an early removal of impurities avoids any unpleasant colour or odour in the final crystals. A multilayer column (diatomaceous earth, activated carbon, cardboard filter) suggested by Wang et al. (2016) is considered as most suitable to improve the effectiveness of this operation. The desalting ion exchange is a mature technology used in erythritol production to remove salts and other ionizable molecules from culture broths. However, considering the demand for regenerant chemicals and the waste produced during regeneration processes (7 times the resin volume (Purolite 2021)), this method should be improved or substituted in order to close loops and move towards a circular economy process. Finally, the primary evaporation removes residual volatile compounds and improves the subsequent chromatographic separation.

The 3rd stage comprises liquid preparative chromatography and secondary evaporation. It was identified as the critical unit operation in the whole erythritol downstream process due to the influence of the preliminary stages on its performance, and its influence on the subsequent secondary concentration and crystallization stage. The selection of the eluent, chromatographic resin and column characteristics (diameter to height ratio, BV), the operational conditions (pH, temperature and flow) and the overload (feed of solution to be treated) are key parameters to be defined during the setting up process. An appropriated overload level (defined as the %BV fed to the column) must be defined to balance the separation efficiency and the demand of energy required in the secondary concentration stage.

The 4th stage comprises crystallization, crystal separation and drying. The most suitable crystallization option is the antisolvent-aided membrane crystallization. This method enables reduced energy requirements for the secondary concentration. Further, the possibility for recovery and recirculation of the antisolvent-aid avoids waste and is a step towards meeting eco-efficiency criteria.

## Opportunities for the production of erythritol in a circular economy context

The circular economy is recognized by the European Union (EU) as an “irreversible, global megatrend” (Friant et al. 2020) based on the idea of the transformation of the industrial production to a sustainable, low-carbon and resource-efficient one. This transformation requires the transition and design of sustainable industrial processes with close-loop and no-waste generation beginning from the selection of the feedstock to the conceptualization of the downstream section (Awasthi et al. 2020; De Angelis 2020). The nature of the substrate used for erythritol production is a key for the sustainable nature of the process. It is necessary to understand the current limitations and challenges raised by renewable feedstocks as discussed in this review and to identify other potential feedstocks to overcome the current use of glucose and glycerol. In addition to the overall goal to decrease the residues produced within the process, it is certainly also essential to identify possible re-uses of any residual streams. Besides, this section presents alternatives for the minimization of the waste produced through the valorization of the residual mother liquor, the recovery of nutrients in the culture broth and the utilization of the biomass.

### Challenges raised by the use of alternative feedstocks

Residual biomass is considered one of the fundamental guarantees of the restoration cycle in a circular economy (Sherwood 2020). Agro-industrial residues as lignocellulosic, agricultural residues and dairy residues are characterized by their high availability and advantageous composition. These residues are a potential source of hexoses, pentoses, glycerol, starch, oligomers and lactose (Cizeikiene et al. 2018; Serna-Loaiza et al. 2019; Louasté and Eloutassi 2020; Omwene et al. 2020).

According to the European Joint Research Centre, the residual biomass represents approximately 442 Mt or 46% of the total agricultural biomass produced in the EU (Camia et al. 2018). The use of these raw materials demands a pretreatment of the substrates during the upstreaming to make them suitable for the production of erythritol. In the case of lignocellulosic biomass, it is necessary to overcome the recalcitrance of the raw material (Daza Serna et al. 2016) and to enhance the sugar recovery (Weinwurm et al. 2012) while minimizing the production of inhibitory compounds such as furfural and 5-(hydroxymethyl)furfural during pretreatment (Weinwurm et al. 2017). Detoxification for removal of inhibitory compounds can be performed by membrane separation, overliming, electrocoagulation or adsorption (Zhou et al. 2013; Haq et al. 2018; Wang et al. 2018; Jeong et al. 2019).

The dairy industry and its derivates contribute approximately 14% to the overall EU agricultural income. The main residue of the dairy industry is whey milk. According to the European Whey Processors Association (EWPA) 4,150,000 tonnes/dry matter of whey milk was produced in 2018 (Carvalho et al. 2013; Hausjell et al. 2019; European Dairy Association 2020). Whey milk is a source of lactose (75–80% dry-wt.), minerals (9–10% dry-wt.; including calcium, sodium, phosphorus, magnesium, potassium, iron, cupper and manganese; (Sebastián-Nicolás et al. 2020)) and proteins (8–11% dry-wt.; including β-lactoglobulin, α-lactoalbumin, bovine serum albumin, immunoglobulins, proteose peptones and minor proteins; Duke and Vasiljevic 2016). The main challenge for the utilization of whey milk in an erythritol manufacturing process is the elimination of the microbiological risk contamination. This can be achieved by technologies such as autoclaving, bactofugation, pasteurization and microfiltration with different consequences for sugar and protein content (Faccia et al. 2013; Hausjell et al. 2019; Wen-qiong et al. 2019).

### Usage of residual mother liquor

The residual mother liquor is a viscous and generally coloured residue from the crystallization stage containing up to 40%wt. of erythritol (Xie et al. 2016). Morioka et al. used this stream as eluent in chromatography separation (Morioka et al. 2000). Wang et al. proposed an approach consisting of the preliminary characterization of by-products in the residual mother liquor (by HPLC, TLC and GC-MS analytics), followed by the screening for a strain able to consume the residual polyols in the mother liquor except erythritol (Wang et al. 2017b). Thus, the yeast *Candida maltose* SJTU82 was found to consume glycerol, ribitol, arabitol, mannitol and residual glucose were in a fed-batch culture. The purified fraction was submitted to ultra- and nanofiltration, decolouration, ion exchange, concentration and crystallization. In this way, it was possible to recover 1 ton of pure erythritol from 5.5 ton of waste mother liquor (Wang et al. 2017b). Through such a valorization approach of the mother liquor, this stream from the concentration stage can be connected to the purification stage to be reprocessed together improving the overall recovery of erythritol.

### Recovery of nutrients

Emerging membrane-based technologies as forward osmosis, membrane distillation and electrodialysis (ED) have been studied for nutrient recovery in wastewater treatment (Xie et al. 2016) and biotechnological processes (Cheryan and Parekh 1995; Shen et al. 2005; Prochaska et al. 2014; Phanthumchinda et al. 2018). With ED, two different types of fractions are obtained: a diluted or deionized fraction with a low, close-to-zero concentration of ions and a second fraction with a high concentration of ions. These ions can be collected as a mixture of ions by using a conventional cationic and anionic membrane configuration (Shen et al. 2006) or as two fractions (base and acid fractions) by using a bipolar membrane configuration (Hab̌ová et al. 2004). The main operational ED conditions include the type of membrane, the flow rate of diluted and concentrated fractions and the voltage or current level (Kresnowati et al. 2019).

ED was used for purification of products and recovery of nutrients from cultivation broths. Hábová et al. reported lactic acid purification by using two stages ED separation. The first ED stage was implemented to concentrate the lactate in solution; the second ED stage using a bipolar membrane was used to recover the lactic acid from the concentrated solution (Hab̌ová et al. 2004). The culture broth was pretreated by ultrafiltration, decolorization and removal of multivalent ions (Ca^+2^, Mg^+2^, Fe^+2^, Zn^+2^) to avoid deposition, fixation of organic material and damage on the membrane surface. The concentrated fraction from the first ED stage increased the concentration level of lactate 4 times compared to the initial. Finally, the second bipolar ED stage allowed to recover 85–98% of the total lactic acid (Hab̌ová et al. 2004). Studies from Cheryan and Parekh as well as Kresnowati and co-workers evaluated the utilization of ED in the separation of glycerol, lactic acid and succinic acid (Cheryan and Parekh 1995) or xylitol (Kresnowati et al. 2019) from a culture broth.

ED might be considered as an alternative for desalination and recovery of nutrients in the recovery stage of the erythritol production. However, further research is needed to determine the concentrations and effect of multivalent ions on the membrane as well as the feasibility of the reutilization of the recovered nutrients.

### Biomass utilization

The physicochemical composition is the key factor for the valorization of the biomass growth during the production of erythritol. Several studies have considered the potential of *Y. lipolytica* in multiple applications including the utilization as an oleaginous platform for obtaining different fatty acids that can be used in biodiesel production (Patel et al. 2016; Howlader et al. 2018)*.* Besides, in 2019, the European Food Safety Authority (EFSA) recognized *Y. lipolytica* as a dietary supplement targeted to a general population from 3 years of age onwards (Turck et al. 2019). Other applications for this yeast include its use as a cell protein source for animal feeding (Bialas et al. 2016).

## Data Availability

Not applicable.
